# Causal relationship between nitrogen dioxide and the risk of Parkinson’s disease: Evidence from a Mendelian randomization study

**DOI:** 10.1097/MD.0000000000042582

**Published:** 2025-05-23

**Authors:** Xingxu Yi, Shasha Song, Zhiqian Cui, Ming Li, Yuxin Huang, Zichen Zhang, Lingmei Kuang, Hong Su

**Affiliations:** a Department of Epidemiology and Health Statistics, School of Public Health, Anhui Medical University, Hefei, Anhui, China; b Inflammation and Immune Mediated Diseases Laboratory of Anhui Province, Hefei, Anhui, China; c Department of Gastroenterology, the Second Hospital of Anhui Medical University, Hefei, Anhui, China; d Center for Big Data and Population Health of IHM, Hefei, Anhui, China.

**Keywords:** causal relationship, Mendelian randomization, nitrogen dioxide, Parkinson’s disease, public health

## Abstract

Several recent observational studies have found associations between nitrogen dioxide (NO_2_) exposure and the risk of Parkinson’s disease (PD), but the causal relationship between them remains unclear. Our objective is to employ a 2-sample Mendelian randomization (MR) approach to determine the causal effect of NO_2_ exposure on the risk of PD. MR analyses were performed using genome-wide association studies (GWAS) data on NO_2_ exposure (n = 456,380) and PD GWAS data (33,674 cases and 449,056 controls). Inverse variance weighting (IVW) was the primary analytical method used to examine causal effects, coupled with the MR-Egger, weighted median, weighted model and MR pleiotropy residual sum and outlier (MR-PRESSO). The main results of the IVW method (odds ratio: 4.701; 95% CI: 1.127–19.615, *P* = .034) showed evidence for a causal relationship between NO_2_ exposure and the risk of PD. Heterogeneity analyses was conducted using the MR-Egger method (Cochran’s *Q* = 1.155; *P* = .764) and IVW (Cochran’s *Q* = 1.356; *P* = .852) demonstrated no statistically significant heterogeneity among the selected SNPs. We employed MR-Egger regression (β intercept = −0.026; SE = 0.058; *P* = .684) and the MR-PRESSO global test (*P *= .840), which revealed no significant impact of pleiotropy on the results of the MR evaluation. Based on MR analysis, higher levels of NO_2_ exposure are causally associated with an increased risk of PD. Consequently, mitigating air pollution could be an important strategy for reducing the risk of PD.

## 1. Introduction

Parkinson’s disease (PD), the second most prevalent age-related neurodegenerative disorder after Alzheimer’s disease, emerges due to the interplay of genetic and environmental factors.^[1]^ It is characterized by motor symptoms such as bradykinesia, muscle rigidity, resting tremors, and postural instability.^[2]^ With the escalating global aging trend, it is projected that PD will impact approximately 14.2 million individuals worldwide by 2040.^[3]^ Studies have shown that living near busy roads and exposure to air pollution are associated with an increased risk of developing PD and dementia. Thus, environmental pollution is also a common risk factor for PD.^[4]^ Although efforts to decrease pollution have led to reduced levels of particulate matter and sulfur dioxide in numerous countries, including China, there has been an upward trend in nitrogen dioxide (NO_2_) levels within urban areas.^[5,6]^ This trend can be attributed to urbanization and the growing number of vehicles, making it a recognized indicator of traffic-related air pollution (TRAP).^[7]^ In recent decades, the harmful effects of air pollution on human health, especially its negative impact on neurodegenerative diseases, have garnered significant attention in the fields of medicine and public health.^[8]^ Therefore, we have a great interest in comprehending the potential cause-and-effect relationship between exposure to NO_2_ and the risk of PD.

Although traditional case-control or cross-sectional research is valuable, it may be difficult to provide accurate results due to the possibility of undetected confounders and reverse causality.^[8,9]^ To overcome this limitation, incorporating genetic differences into risk assessments for environmental pollution can provide a comprehensive understanding of the health hazards associated with environmental exposure for specific individuals.^[10,11]^ Mendelian randomization (MR) makes use of the random distribution of human genetic variants within a population to circumvent the impact of confounding variables.^[12]^ The primary objective of this study was to delve into the causal connection between NO_2_ exposure and the risk of PD, employing the MR method. The result will contribute to the identification of specific environmental risk factors for PD and offer important guidance for implementing targeted public health strategies in an aging society.

## 2. Methods

### 2.1. Study design and data sources

**Figure 1.**
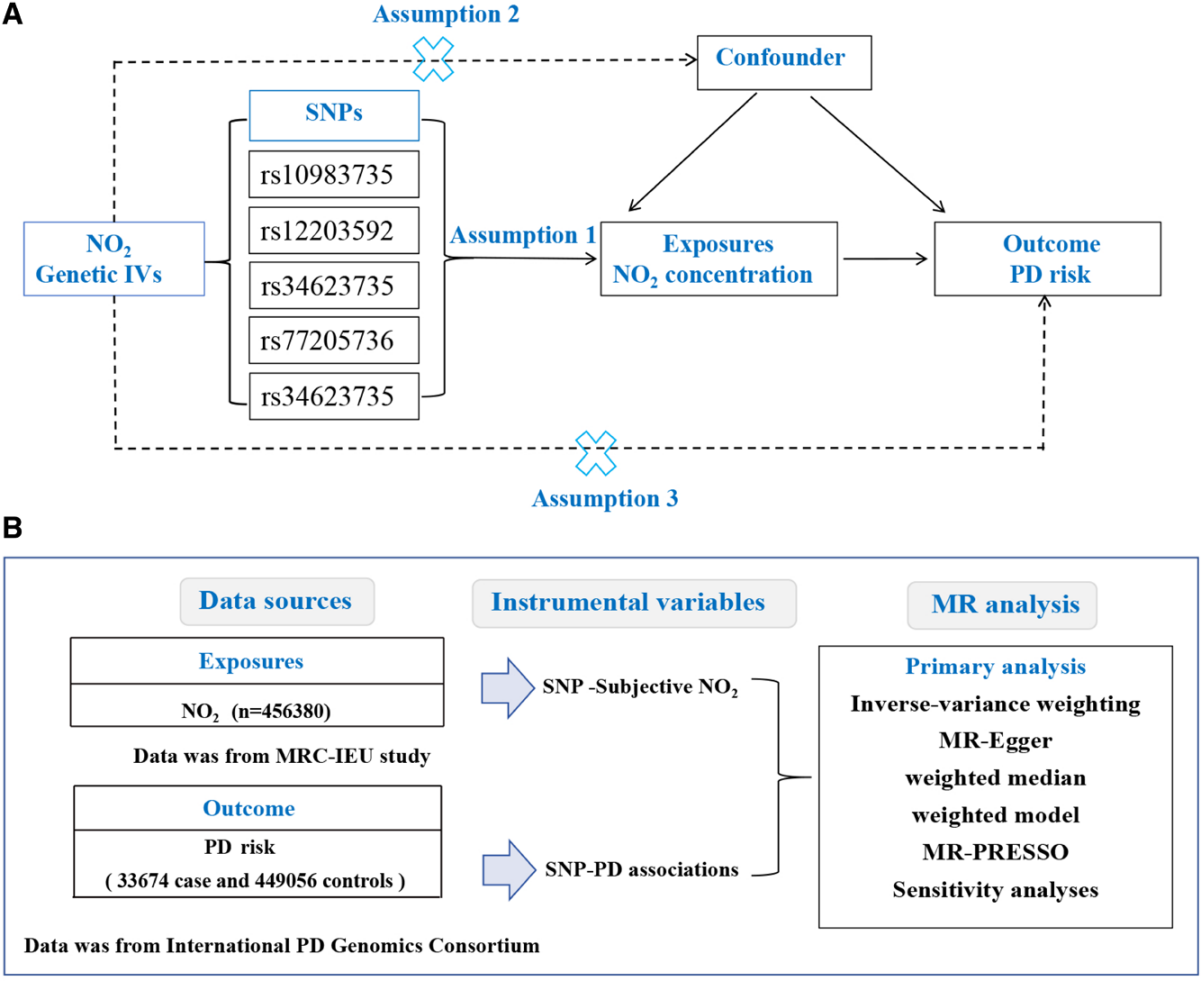
Summary of data sources and flowchart of study design. Study design (A), data sources (B).

MR study design is analogous to a randomized controlled trial (RCT), leveraging the random allocation of parental alleles during gamete formation to minimize the impact of confounding factors and reverse causality.^[13]^ Following this principle, we utilize single nucleotide polymorphisms (SNPs) as instrumental variables (IVs) to deduce causality, integrating summary statistics from existing genome-wide association studies (GWAS). The criteria and process for selecting SNPs as IVs were as follows:^[14]^ relevance: SNPs significantly associated with NO₂ exposure (*P* < 5 × 10^−8^) were extracted from GWAS summary statistics. To ensure sufficient instrument strength, only SNPs with *F*-statistics > 10 were retained. Independence: we performed linkage disequilibrium (LD) clumping (*r*^2^<0.001, clumping distance = 10,000 kb) to eliminate SNPs in linkage disequilibrium. Exclusion restriction: Horizontal pleiotropy was assessed via MR-Egger intercept tests (*P *> .05). As illustrated in Figure 1A.

We used GWAS data on NO_2_ exposure from the MRC Integrated Epidemiology Unit (MRC-IEU) study at the University of Bristol (n = 456,380) and PD GWAS data from the International PD Genomics Consortium (33,674 cases and 449,056 controls), as depicted in Figure 1B. In order to minimize potential biases arising from population stratification, only individuals of European ancestry were included in both the exposure (dataset: ukb-b-2618) and outcome (dataset: ieu-b-7) cohorts of the study (https://gwas.mrcieu.ac.uk/). This study utilized publicly available data. As the analysis did not involve any individual-level data or new human participants, neither informed consent nor ethical approval was required.

### 2.2. Selection of IVs

We employed the following quality control steps to select appropriate IVs, ensuring the accuracy of causal inference regarding the association between NO_2_ exposure and PD risk. First, we used the genome-wide significance threshold (*P *< 5 × 10^−8^) to select SNPs that showed strong associations with NO_2_ exposure.^[15]^ These SNPs were subsequently selected as potential IVs. Second, SNPs with a minor allele frequency (MAF) of ≤0.01 were excluded to avoid issues related to rare variants. To avoid bias from LD, we performed LD clumping (*R*^2^ < 0.001, clumping window = 10,000 kb) and retained only the SNP with the lowest *P*-value in each clump.^[16]^ Third, the strength of the correlation between IVs and exposure was estimated using the *F*-statistic: *F* = *R*^2^ (n − *k* − 1)/*k*(1 − *R*^2^), where *k* is the number of IVs, n is the sample size, and *R*^2^ is the exposure variance explained by the chosen SNPs.^[17]^ The *R*^2^ value was calculated using the formula 2 × MAF(1-MAF) beta^2^, where beta represents the effect estimate of the genetic variant in the exposure, measured in standard deviation units. An *F*-statistic > 10 for the chosen SNPs indicates that they are strong IVs. Moreover, we assessed the association between the selected SNPs and potential confounding factors using PhenoScanner database to identify and exclude any SNPs that had significant associations with these confounders. Fourth, we verified whether the effect of SNPs on exposure was consistent with the effect of the same allele on the outcome and removed palindromic SNPs from the IVs.^[18]^ Fifth, to ensure that the selected IVs affected the outcome only through the exposure and to detect any potential horizontal pleiotropy, we applied MR-Egger regression and the MR pleiotropy residual sum and outlier (MR-PRESSO) method.^[19]^ These analyses allowed us to identify and remove outliers that could be indicative of pleiotropic effects, thereby providing a corrected estimate of the causal effect.

### 2.3. Statistical analysis

In the realm of MR method selection, it is acknowledged that the IVW approach is notably reliable when neither heterogeneity nor pleiotropy affects the data. This method eschews the influences of confounders, providing more precise estimates.^[20]^ Our research chiefly harnesses the IVW method to delve into the causal relationships, utilizing a weighted linear regression to gauge the mean influence of genetic variables on causality. To bolster our analysis, we integrated additional techniques including Weighted Median, Weighted Model, MR-Egger, MR-PRESSO to evaluate the collateral link between NO_2_ exposure and PD risk. MR-Egger regression, alongside the MR-PRESSO global test, was deployed to ascertain any broad pleiotropic impacts of the included SNPs. We also applied the MR-PRESSO outlier test for spotting outliers indicative of pleiotropic biases, especially since MR-Egger regression’s diminished statistical sensitivity and power might overlook them.^[21,22]^ The weighted model presumes validity in 50% of the genetic instruments’ information.^[23]^ In contrast, MR-Egger regression, even with all instruments potentially being invalid, can still produce unbiased estimates as long as the variants uphold the instrument strength independent of direct effects (InSIDE) criterion.^[19]^ To scrutinize the heterogeneity across selected SNPs, Cochran’s *Q* statistic was employed.^[24]^ Furthermore, a sensitivity analysis, excluding 1 SNP at a time, was conducted to identify any significantly influential SNPs, thus verifying the reliability and consistency of our findings regarding causal impacts. For statistical inference, a 2-tailed test determined *P*-values, with significance set at a *P*-value lower than .05. The odds ratios (OR) with 95% confidence intervals (95% CI) were used to quantitatively describe the association between exposures and outcome. All statistical analyses were carried out using R software (version 4.3.0).

## 3. Results

### 3.1. *Extracted genetic IVs related to NO*_*2*_

We identified 5 independent SNPs (rs10983735, rs12203592, rs34623735, rs7225402, and rs77205736) as IVs for NO₂ exposure, all reaching genome-wide significance (*P* < 5 × 10^−8^). As detailed in Table 1, these SNPs exhibited features consistent with valid instruments, including chromosomal locations distinct from known pleiotropic regions. The instrument strength was rigorously evaluated through *F*-statistics derived from summary data. As depicted in Table S1, Supplemental Digital Content, https://links.lww.com/MD/O995, each SNP exhibited strong predictive power, with *F*-statistics over 10 (range: 33.273–46.111; mean: 38.370), indicating a minimal risk of weak instrument bias in our MR analysis.

**Table 1:** Associations of single nucleotide polymorphisms with nitrogen dioxide and Parkinson’s disease.

SNPs	Chromosome	Effect alleles	Other alleles	NO_2_			PD		
				Beta	SE	*P*-value	Beta	SE	*P*-value
rs10983735	9	A	G	0.016	0.003	5.90 × 10^−09^	0.026	0.031	.395
rs12203592	6	T	C	0.016	0.002	3.60 × 10^−11^	0.021	0.026	.411
rs34623735	10	T	C	0.013	0.002	3.70 × 10^−09^	0.005	0.019	.796
rs7225402	17	C	T	−0.025	0.004	8.00 × 10^−09^	−0.041	0.041	.321
rs77205736	8	T	C	0.015	0.002	1.10 × 10^−11^	0.045	0.024	.065

NO_2_ = nitrogen dioxide, PD = Parkinson’s disease, SNPs = single nucleotide polymorphisms.

### 3.2. *Pleiotropy and heterogeneity analysis*

To assess horizontal pleiotropy, we performed tests to explore potential gene pleiotropy among the selected SNPs. The intercept term of the MR-Egger regression (β intercept = −0.026; *SE *= 0.058; *P *= .684) and the MR-PRESSO global test (*P *= .840) indicated no significant evidence of pleiotropy. In addition, no anomalous variations were detected in the MR-PRESSO outlier test. These findings indicate that the 5 SNPs do not affect PD through biological pathways independent of NO_2_ exposure. Furthermore, to assess the heterogeneity among the selected SNPs, we utilized Cochran’s *Q* statistic. The results indicated no significant heterogeneity among the selected SNPs for MR-Egger (Cochran’s *Q *= 1.155; *Q*_df = 3; *P* = .764) and IVW (Cochran’s *Q* = 1.356; *Q*_df = 4; *P* = .852) methods. These results suggest that the 5 genetic variants of NO_2_ did not show significant heterogeneity in the PD GWAS dataset. As a result, we can utilize the 5 NO_2_ exposure genetic variants that we have chosen as efficient IVs for a 2-sample MR study. The detailed information is shown in Table 2.

**Table 2:** Pleiotropy and heterogeneity test of nitrogen dioxide genetic instrumental variables for Parkinson’s disease.

Pleiotropy test				Heterogeneity test					
MR-egger			PRESSO	MR-egger			IVW		
Intercept	SE	*P*-value	*P*-value	*Q*	*Q*_df	*P*-value	*Q*	*Q*_df	*P*-value
−0.026	0.058	.684	.840	1.155	3	.764	1.356	4	.852

IVW = inverse variance weighted, MR = Mendelian randomization.

### 3.3. *MR analysis of NO*_*2*_ *level and PD*

**Figure 2.**
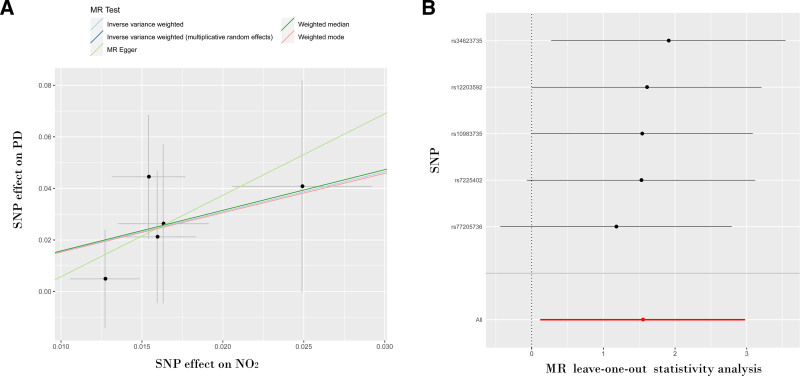
Scatter plot and leave-one-out sensitivity analysis for the causal effect between nitrogen dioxide on PD risks. Scatter plot (A), sensitivity analysis (B). PD = Parkinson’s disease.

The IVW analysis (OR: 4.701; [95% CI: 1.127–19.615], *P* = .034) provides substantial evidence indicating a causal link between NO_2_ exposure and an increased risk of PD. Meanwhile, no statistically significant findings were observed using the MR-Egger, weighted median, and weighted model methods, as presented in Table 3 and Figure 2A. Ideally, achieving significant results across all 3 strategies enhances the robustness of the findings. However, the IVW method, when juxtaposed with other MR techniques, especially MR-Egger, is distinguished by its superior statistical potency.^[25]^ According to the established benchmarks for validating MR findings, outcomes derived from the IVW technique were significant, with neither pleiotropy nor heterogeneity observed. Even in circumstances where alternative methods failed to reach statistical significance, their outcomes were nevertheless regarded as affirmative, provided their Beta-values consistently aligned in the same direction.^[26]^ MR forest plots and funnel plots were used to evaluate the effect of NO_2_ exposure on the risk of PD, as shown in Figure S1, Supplemental Digital Content, https://links.lww.com/MD/O995.

**Table 3:** Mendelian randomization analysis results between nitrogen dioxide and Parkinson’s disease.

Exposure	Outcome	Methods	MR analyses of NO_2_ and the risk of PD			
			No. of SNPs	OR	95% CI	*P*-value
NO_2_	PD	IVW	5	4.701	(1.127–19.615)	.034
		MR-Egger	5	23.569	(0.018–31218.161)	.452
		Weighted median	5	4.833	(0.828–28.228)	.080
		Weighted mode	5	4.606	(0.406–52.200)	.285

CI = confidence interval, IVW = inverse variance weighted, NO_2_ = nitrogen dioxide, OR = odds ratio, PD = Parkinson’s disease, SNPs = single nucleotide polymorphisms.

Then we employed a reverse MR approach, utilizing PD SNPs as IVs to investigate the effects of these SNPs on NO_2_. Summary statistics were directly extracted from a GWAS involving individuals of European ancestry, focusing on 23 validated SNPs, as presented in Table S2, Supplemental Digital Content, https://links.lww.com/MD/O995. These SNPs exhibited a strong correlation with PD at a significance level of *P *< 5 × 10^−8^. Despite employing various analytical methods, the result did not provide any statistically significant evidence of a causal relationship between genetically predicted PD and NO_2_, as presented in Table S3, Supplemental Digital Content, https://links.lww.com/MD/O995.

### 3.4. Sensitivity analyses

Sensitivity analyses were conducted using the leave-one-out method, which provided consistent outcomes, as shown in Figure 2B. The results show that higher levels of NO_2_ exposure are positively and causally associated with an increased risk of PD.

## 4. Discussion

This study provided new evidence for evaluating the impact of environmental pollutants on the risk of PD, enabling the targeted implementation of public health interventions, and thereby contributing to the prevention and delayed onset of the disease. Despite the lack of effective clinical treatments for PD, the decades-long prodromal period before clinical symptoms manifest offers a potential window for intervention.^[27,28]^ Unlike genetic factors, which cannot be changed, air pollution can be effectively reduced through targeted interventions. By enhancing air quality, it may help delay the pathological progression of PD and reduce its risk of onset, thereby enhancing the overall quality of life for high-risk populations.

As the global population ages and average life expectancy rises, the prevalence and incidence of PD increase nearly exponentially with age, peaking beyond the age of 80.^[29]^ Coincidentally, due to the acceleration of global industrialization and urbanization, especially in urban areas, motor vehicle exhaust has become a major source of air pollutants.^[30,31]^ These pollutants greatly affect the composition of the atmosphere and pose serious consequences for both climate and human health. The association between NO_2_, as an indicator of TRAP, and the risk of PD has attracted widespread attention. However, previous observational studies have reported an inconsistent association between exposure to NO_2_ and the risk of PD. In a national longitudinal study on the health effects of air pollution, particulate matter 10 was found to significantly impact PD, whereas 9 other pollutants, including NO_2_, had no significant effects.^[8]^ Conversely, a recent retrospective cohort study in JAMA has reignited broad interest.^[32]^ The study analyzed data from 78,830 Seoul residents and found a significant link between higher NO_2_ exposure and an increased risk of PD, particularly at levels above 0.038 ppm. Importantly, the association remained robust even after adjusting for covariates and conducting sensitivity analyses with a 1-year lag. The discrepancies in the findings could be attributed to the complexity of air pollutants, which consist of various components like gases, particulate matter, organic compounds, and metals found within air pollution.^[33]^ These intricate mixture of air pollutants and the constantly changing nature of their components make it challenging to discern the precise compounds involved and their potential causal relationships with specific diseases. Additionally, discrepancies in study design, exposure time to air pollutants, measurement methods, and adjustments for other variables across different studies may also contribute to the inconsistent findings.^[8,34]^ As a result, observational studies can be influenced by biases stemming from reverse causation and residual confounding. To overcome these limitations, an MR approach can be used, which takes into account the genetic variation that may influence an individual’s susceptibility to a particular pollutant and uses genetic polymorphisms as IVs to assess the causal effect of “exposure-outcome.”^[35]^ Based on this, it enables a more personalized and precise assessment of the risk of environmental impacts on health. As expected, our study yielded reliable causal inferences, confirming the crucial role of NO_2_ in the occurrence of PD.

While the exact mechanism by which NO_2_ increases the risk of PD remains uncertain, there are several potential explanations. First, the neuropathological features of PD, including the loss of dopaminergic neurons, the formation of Lewy bodies and neurons due to ɑ-synuclein aggregation, and the presence of neuroinflammation in the brain.^[1,36]^ Air pollutants, including NO_2_, NO_*x*_, O_3_, and particulate matter, have the capability to cause chronic inflammatory processes in the respiratory tract and to disrupt both the nasal/olfactory epithelial barrier and the blood-brain barrier. This leads to intracerebral neurodegeneration and the development of inflammatory markers such as α-synuclein and amyloid β-42.^[37,38]^ Second, the high oxidation potential and activity of NO_2_ itself may contribute to the accumulation of reactive oxygen species and oxidative damage to lipids, proteins, DNA, and RNA. This compromises neuronal function and structural integrity, thereby accelerating the pathological progression of PD.^[39]^ Compared to other pollutants, it has a more pronounced effect on cell and tissue oxidative damage. Third, NO_2_ also participates in photochemical reactions, particularly when interacting with sunlight, resulting in the formation of other harmful oxidizing chemicals like O_3_. These chemicals can cause additional oxidative stress and cell damage.^[40]^ Fourth, NO_2_ is produced mainly from combustion processes, such as transportation and coal combustion, and is usually concentrated in urban and traffic-intensive areas, leading to increased levels of NO_2_ in urban areas. This trend can be attributed to urbanization and the rising number of vehicles.^[5]^ In conclusion, the oxidative potential of NO_2_ and its interaction with other pollutants confer unique significance to its role in the progression of PD. This is especially pertinent for urban residents and individuals residing in traffic-congested regions, where the control of NO_2_ emissions plays a pivotal role in safeguarding health. Improving air quality and reducing NO_2_ exposure during the prodromal phase of PD may help mitigate neurodegenerative pathological processes and delay disease progression. The mechanisms discussed above provide a partial explanation for the observed association between elevated NO_2_ levels and increased PD risk. Similarly, our results also support the aforementioned viewpoint.

Our study possesses several strengths. We analyzed the association between NO_2_ and PD using the MR technique, providing new evidence for evaluating the impact of environmental pollutants on PD risk. Compared to observational studies, MR methods are less susceptible to confounding and reverse causality. Additionally, we conducted outlier assessments and extensive sensitivity analyses to account for various patterns of pleiotropy, thereby enhancing the robustness of our results. For healthcare professionals, our findings underscore the necessity of integrating a patient’s environmental exposure history into the clinical assessment of PD. Incorporating cumulative nitrogen dioxide exposure data into the PD screening process is expected to identify individuals at an increased risk of developing the disease due to prolonged exposure to polluted environments. This early identification provides an opportunity to implement timely preventive measures and intervention strategies that have the potential to slow disease progression and reduce the health burden associated with PD.

Several limitations of this study should be noted, despite the meaningful results produced by the MR method. Since the number of IVs fulfilling the strict threshold (*P* < 5 × 10^−8^) was extremely small, this poses 2 main limitations. First, fewer IVs reduce statistical power to detect causal effects, particularly for exposures with smaller effect sizes. Second, despite strong instrument strength (*F*-statistics > 10 for all IVs), the small SNP set leads to wider CIs in causal estimates. Notably, with only 5 IVs, methods like MR-PRESSO may have limited power to detect horizontal pleiotropy, as outlier detection requires sufficient SNPs to distinguish true causal effects from bias. Future studies could strengthen causal inference by utilizing expanded GWAS datasets to identify additional valid IVs.

Although our study offers valuable insights into the causal relationship between exposure and outcome, the sample population was predominantly of European ancestry due to data source limitations. Consequently, the generalizability of our findings to other racial and ethnic groups may be limited. Genetic variations and their associations with traits can vary across populations due to genetic heterogeneity and population-specific evolutionary history. To enhance the external validity of our results, we recommend that future studies replicate this analysis in diverse ethnic groups (e.g., East Asian populations) and geographically distinct regions.

Furthermore, we utilized publicly available summary statistics from the MRC-IEU GWAS database, selected based on specific disease criteria. The absence of individual genotype data restricted our ability to conduct detailed population stratification analyses. To enhance the validity of our research findings, we recommend that future research should prioritize the use of cohort populations such as the UK Biobank. By employing genetic risk score-based single-sample MR analyses and controlling for principal components, this can minimize the potential impact of population stratification. Moreover, the adoption of advanced MR methods, such as MR-PRESSO or MR-RAPS (Robust Adjusted Profile Score), could provide additional robustness against residual population stratification and horizontal pleiotropy, while still aligning with traditional MR estimates.

## 5. Conclusions

Our research suggested that NO_2_ exposure and the risk of PD are causally related in a positive way. This finding could potentially serve as a guide for implementing targeted public health measures, which could be crucial in efforts to prevent or delay the onset of PD in older individuals and in regions with high levels of NO_2_ exposure..

## Acknowledgments

We thank the IEU OpenGWAS databases for providing the data platform and the authors of the original GWAS datasets.

## Author contributions

**Conceptualization:** Hong Su.

**Data curation:** Xingxu Yi, Shasha Song, Zhiqian Cui, Zichen Zhang, Lingmei Kuang.

**Formal analysis:** Xingxu Yi.

**Funding acquisition:** Hong Su.

**Investigation:** Yuxin Huang.

**Methodology:** Xingxu Yi, Shasha Song, Ming Li, Zichen Zhang.

**Project administration:** Hong Su.

**Software:** Zhiqian Cui, Ming Li.

**Supervision:** Hong Su.

**Visualization:** Zhiqian Cui, Yuxin Huang.

**Writing – original draft:** Xingxu Yi.

**Writing – review & editing:** Zichen Zhang, Lingmei Kuang, Hong Su.

## Supplementary Material


