# Supplementary material for: Causal relationship between nitrogen dioxide and the risk of Parkinson’s disease: Evidence from a Mendelian randomization study
Source: Medicine (Baltimore). 2025 May 23;104(21):e42582. doi: 10.1097/MD.0000000000042582 (PMC12114015; doi:10.1097/MD.0000000000042582)
Supplement: Supplementary file 1 [file medi-104-e42582-s001.pdf]

Supporting Information

Causal relationship between Nitrogen dioxide and the risk of Parkinson’s Disease: evidence from a Mendelian randomization study

Xingxu Yi, MS [a,b], Shasha Song, MS [a,b,d], Zhiqian Cui, MS [a,b], Ming Li, MS [a,b], Yuxin Huang, MS [a,b], Zichen Zhang, MS [a,b], Lingmei Kuang, MS [a,b], Hong Su, PhD [a,b,c*iD]

a Department of Epidemiology and Health Statistics, School of Public Health, Anhui Medical University, Hefei, Anhui, 230032, China.

b Inflammation and Immune Mediated Diseases Laboratory of Anhui Province, Hefei, Anhui, 230032, China.

c Center for Big Data and Population Health of IHM, Hefei, Anhui, 230032, China.

d Department of Gastroenterology, the Second Hospital of Anhui Medical University, Hefei, Anhui, 230032, China.

Corresponding authors:

Full name: Hong Su*

E-mail address: 271244914@qq.com.

Postal address: Anhui Medical University, 81 Meishan Road, Hefei, Anhui, 230032, China

*

Tables legends

Table S1 F-statistics for the five instrumental variables.

Table S2 Associations of single nucleotide polymorphisms with Parkinson’s disease and Nitrogen dioxide.

Table S3 Reverse Mendelian randomization analysis results between Parkinson’s disease and Nitrogen dioxide.

Figure legends

Figure S1. Mendelian randomization forest plot and funnel plot evaluation of risk to Nitrogen dioxide and Parkinson’s disease.

Table S1 F-statistics for the five instrumental variables.

SNPs	F-statistic
rs10983735	33.867
rs12203592	43.826
rs34623735	34.775
rs7225402	33.273
rs77205736	46.111
Mean	38.370

*SNPs = Single-nucleotide polymorphisms.

Table S2 Associations of SNPs with Parkinson's disease risk and Nitrogen dioxide.

SNPs	Chromosome	Effect alleles	Other alleles	PD			NO_2		
				Beta	SE	*P value*	Beta	SE	*P value*
rs10451230	17	T	A	-0.096	0.018	4.42E-08	-0.003	0.002	0.160
rs10513789	3	G	T	-0.160	0.022	3.18E-13	-3.56E-04	0.003	0.890
rs10847864	12	T	G	0.127	0.018	9.81E-13	-0.008	0.002	8.70E-05
rs12934900	16	T	A	0.122	0.018	4.33E-11	-0.003	0.002	0.150
rs144814361	10	T	C	0.441	0.068	9.07E-11	-0.012	0.008	0.130
rs329647	11	C	G	-0.113	0.018	1.94E-10	-0.003	0.002	0.220
rs34311866	4	C	T	0.227	0.023	7.97E-23	0.001	0.003	0.590
rs35265698	6	G	C	-0.200	0.030	3.93E-11	-0.002	0.003	0.350
rs356203	4	T	C	-0.240	0.018	3.01E-41	9.61E-04	0.002	0.650
rs35749011	1	A	G	0.751	0.066	5.02E-30	-0.011	0.009	0.200
rs4488803	3	A	G	-0.114	0.020	1.08E-08	-7.93E-04	0.002	0.710
rs4588066	18	A	G	0.105	0.018	4.45E-09	0.003	0.002	0.16
rs4613239	2	G	C	0.178	0.025	6.21E-13	0.006	0.003	0.051
rs4698412	4	A	G	0.126	0.017	7.05E-14	2.72E-04	0.002	0.890
rs4774417	15	A	G	0.105	0.019	4.63E-08	-0.001	0.002	0.550
rs58879558	17	C	T	-0.238	0.025	1.36E-21	-0.005	0.002	0.029
rs620490	8	G	T	-0.117	0.019	6.46E-10	-9.17E-04	0.002	0.680
rs6741007	2	G	T	-0.123	0.018	2.09E-12	3.07E-04	0.002	0.880
rs75505347	12	T	C	0.392	0.067	6.12E-09	0.004	0.008	0.660
rs75646569	5	G	T	0.192	0.027	5.62E-13	-9.93E-04	0.003	0.780
rs7695720	4	C	A	-0.126	0.021	1.53E-09	9.71E-04	0.002	0.690
rs823106	1	C	G	-0.149	0.024	4.10E-10	-0.006	0.003	0.070

SNPs = Single-nucleotide polymorphisms, NO_2 =Nitrogen dioxide, PD = Parkinson's disease.

Table S3 Reverse Mendelian randomization analyses results between Parkinson's disease risk and Nitrogen dioxide.

Methods	MR analyses		
	No. of SNPs	OR (95% CI)	*P value*
IVW (multiplicative random effects)	23	1.000(0.992-1.010)	0.806
MR Egger	23	1.000(0.977-1.023)	0.982
Weighted median	23	1.000(0.993-1.012)	0.655
Weighted mode	23	1.000(0.985-1.015)	0.989

SNPs = single-nucleotide polymorphisms, NO_2 =Nitrogen dioxide, PD = Parkinson's disease, IVW = inverse variance weighted, OR = odds ratio, CI = confidence interval.

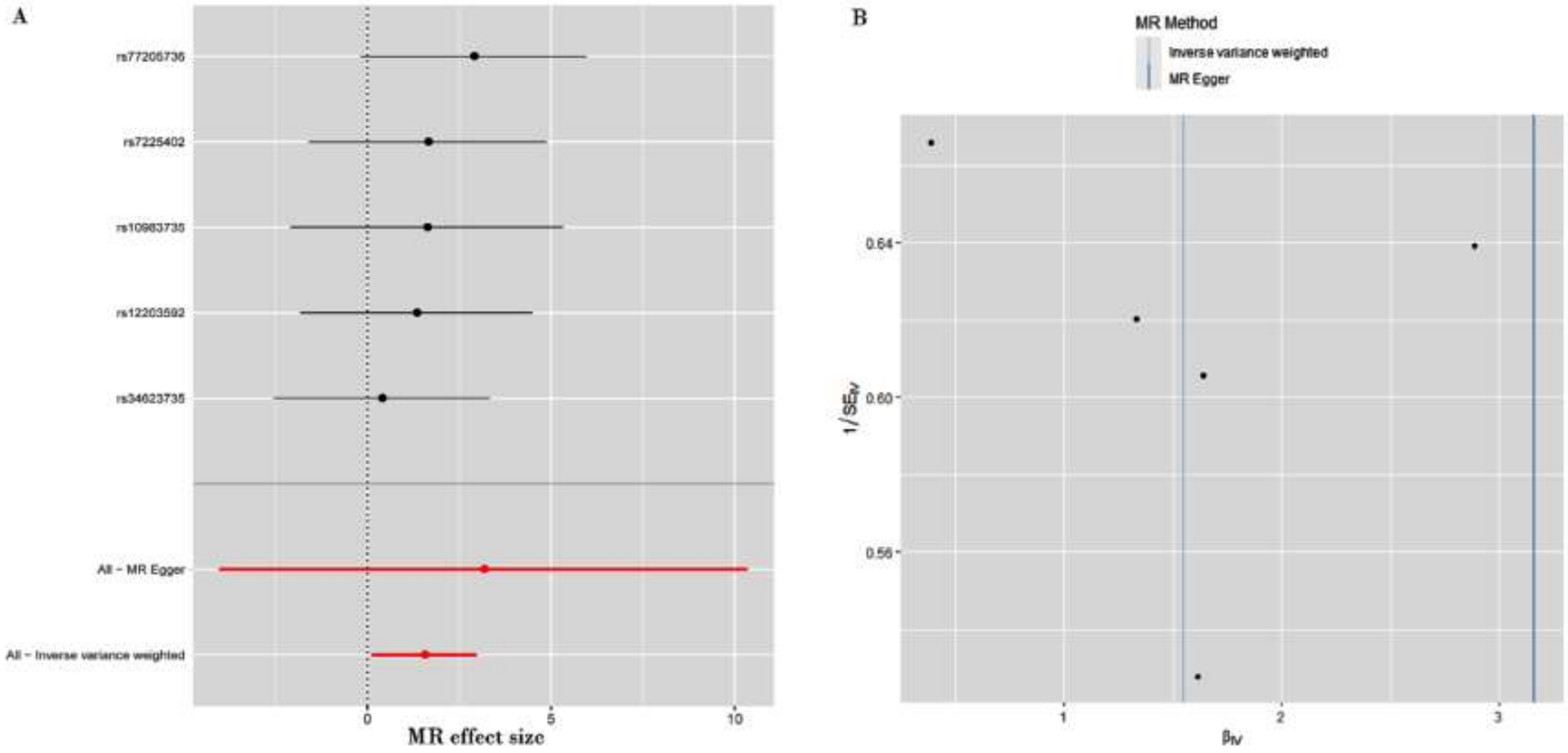

Figure S1 MR Forest plot and funnel plot evaluation of risk to Nitrogen dioxide and Parkinson's disease. Forest plot (A), funnel plot (B).
